# The Three Musketeers in Cancer Therapy: Pharmacokinetics, Pharmacodynamics and Personalised Approach

**DOI:** 10.3390/jpm15110516

**Published:** 2025-10-31

**Authors:** Milan Zarić, Petar Čanović, Radica Živković Zarić, Simona Protrka, Miona Glišić

**Affiliations:** 1 Department of Biochemistry, Faculty of Medical Sciences, University of Kragujevac, Svetozara Markovića 69, 34000 Kragujevac, Serbia; petar.c89@gmail.com; 2 Department of Pharmacology and Toxicology, Faculty of Medical Sciences, University of Kragujevac, Svetozara Markovića 69, 34000 Kragujevac, Serbia; radica_zivkovic@yahoo.com; 3 Faculty of Medical Sciences, University of Kragujevac, Svetozara Markovića 69, 34000 Kragujevac, Serbia; 4 Department of Dentistry, Faculty of Medical Sciences, University of Kragujevac, Svetozara Markovića 69, 34000 Kragujevac, Serbia; mionagrujovic@yahoo.com

**Keywords:** personalized medicine, pharmacokinetics, pharmacodynamics, biomarkers, targeted therapy, cancer therapy

## Abstract

Cancer therapy is rapidly evolving from a one-size-fits-all paradigm toward highly personalized approaches. Traditional chemotherapies and radiotherapies, while broadly applied, often yield suboptimal outcomes due to tumor heterogeneity and are limited by significant toxicities. In contrast, precision oncology tailors prevention, diagnosis, and treatment to the individual patient’s genetic and molecular profile. Key advancements underscore this shift: molecularly targeted drugs (e.g., trastuzumab for HER2-positive breast cancer, EGFR and ALK inhibitors for lung cancer) have improved efficacy and reduced toxicity compared to conventional therapy. Pharmacokinetic (PK) and pharmacodynamic (PD) considerations are central to personalizing treatment, explaining variability in drug exposure and response among patients and guiding dose optimization. Modern strategies like therapeutic drug monitoring and model-informed precision dosing seek to maintain drug levels in the therapeutic range, improving outcomes. Immunotherapies, including checkpoint inhibitors and CAR-T cells, have transformed oncology, though patient selection via biomarkers (such as PD-L1 expression or tumor mutational burden) is critical to identify likely responders. Innovative drug delivery systems, notably nanomedicine, address PK challenges by enhancing tumor-specific drug accumulation and enabling novel therapeutics. Furthermore, rational combination regimens (informed by PK/PD and tumor biology) are being designed to achieve synergistic efficacy and overcome resistance. Key barriers include the high cost of biomarker testing, insufficient laboratory infrastructure, and inconsistent reimbursement policies. Operational inefficiencies such as long turnaround times or lack of clinician awareness further limit the use of precision diagnostics. Regulatory processes also remain complex, particularly around the co-development of targeted drugs and companion diagnostics, and the evidentiary requirements for rare subgroups. Addressing these barriers will require harmonized policies, investment in infrastructure, and educational initiatives to ensure that the promise of personalized medicine becomes accessible to all patients. Ensuring that advances are implemented responsibly—guided by pharmacological insights, supported by real-world evidence, and evaluated within ethical and economic frameworks—will be critical to realizing the full potential of personalized cancer medicine.

## 1. Introduction

Cancer continues to be a leading cause of morbidity and mortality worldwide, posing significant challenges to global health systems [1]. Traditional treatment modalities, such as chemotherapy and radiotherapy, often follow a “one-size-fits-all” approach, applying uniform regimens across diverse patient populations. This strategy frequently results in suboptimal outcomes, as tumors with distinct molecular profiles may respond differently to the same therapy. Furthermore, these conventional treatments are associated with considerable off-target toxicities, which can limit dosing and negatively impact patients’ quality of life.

Interpatient variability in drug metabolism, genetic mutations, and tumor microenvironment further contributes to inconsistent treatment responses. Consequently, many patients experience either insufficient therapeutic benefit or severe adverse effects. These limitations underscore the urgent need for more individualized strategies that consider both the patient’s biology and the tumor’s molecular characteristics. Personalized medicine is becoming central in cancer prevention, diagnosis, prognosis, and treatment. Its clinical value is evident in the adoption of molecularly targeted therapies that improve efficacy while reducing toxicity. Genetic predisposition testing (e.g., BRCA mutations in breast cancer) enables risk assessment, early screening, and preventive strategies. Advances in tumor profiling have revealed molecularly distinct cancer subtypes requiring tailored therapies, such as trastuzumab for HER2-positive (human epidermal growth factor receptor 2 positive) breast cancer, cetuximab for KRAS wild-type colorectal cancer, tyrosine kinase inhibitors for chronic myeloid leukemia (CML), gastrointestinal stromal tumors, and non-small-cell lung cancer (NSCLC), and agents like vemurafenib or olaparib in melanoma, ovarian, breast, and prostate cancers. These successes highlight a shift toward treatment decisions guided by tumor molecular signatures rather than tissue of origin, ultimately improving prognosis and quality of life [2,3,4,5].

The primary aim of this review is to highlight several recent innovations in cancer therapy within a single comprehensive article. Given the rapid pace of progress in oncology, it is impossible to cover every emerging development; therefore, this work focuses on selected advancements with substantial clinical relevance [6,7,8,9]. Particular emphasis is placed on the roles of pharmacokinetics and pharmacodynamics, which are critical for understanding interindividual variability in drug response and optimizing therapeutic regimens [10,11]. Additionally, the review underscores the importance of a personalized approach, tailoring treatment strategies to the unique molecular and physiological characteristics of each patient with diagnosed cancer. By integrating these principles, clinicians can enhance therapeutic efficacy, reduce toxicity, and ultimately improve patient outcomes, providing a concise overview of the current trends and novelties shaping precision oncology [12,13,14,15,16].

## 2. Pharmacokinetics and Pharmacodynamics in the Context of Personalized Cancer Therapy

Modern personalized oncology is built on the understanding that each patient possesses a unique constellation of genetic, biochemical, and physiological traits that profoundly influence the effects of cancer therapies. Two fundamental disciplines underpin the ability to tailor treatments effectively: pharmacokinetics (PK), which describes how a drug is absorbed, distributed, metabolized, and eliminated by the body, and pharmacodynamics (PD), which examines how the drug interacts with molecular targets to produce biological effects. Considering PK and PD together enables clinicians and researchers to explain the variability in treatment outcomes and to refine both drug selection and dosing to maximize therapeutic benefit while minimizing toxicity [1,2,3,4,17,18].

### 2.1. Pharmacokinetics and Interindividual Variability

Considerable differences in systemic drug exposure are commonly observed among patients receiving identical anticancer regimens, and these differences are largely dictated by PK factors [5,6]. Contributing elements include inherited variations in drug-metabolizing enzymes, polymorphisms in membrane transporters, age-related physiological changes, coexisting diseases, and the functional status of organs such as the liver and kidneys. Consequently, some patients may accumulate drugs to toxic levels, whereas others may receive subtherapeutic exposure and exhibit inadequate responses [7,8].

The cytochrome P450 family metabolises many oral targeted anticancer drugs [9,10]. Genetic variants affecting these enzymes can substantially alter drug clearance. For example, CYP3A5, a key enzyme in the CYP3A subfamily, is expressed in only a subset of individuals (those carrying at least one 1 allele). In patients treated with the multitargeted TKI sunitinib, 1 allele carriers (CYP3A5 expressors) showed faster metabolism resulting in a need for more frequent dose reductions due to toxicity [19,20]. Conversely, the loss-of-function CYP3A53 allele (homozygous in 3/3 patients) is associated with slower clearance; this can elevate drug exposure and potentially increase toxicity or efficacy depending on the therapeutic window. Similarly, a reduced-function CYP3A422 variant has been linked to higher plasma levels of several drugs (e.g., the breast cancer therapy exemestane) [21], suggesting that CYP3A4 polymorphism may also heighten exposure (and toxicity risk) for TKIs (Tirosine Kinase Inhibitors) predominantly cleared by CYP3A4.

Polymorphisms in other CYPs can influence specific targeted drugs. CYP2D6, although not a major pathway for most TKIs, plays a role in metabolising the EGFR inhibitor gefitinib [22]. Clinical studies have shown that patients with reduced CYP2D6 function (poor or intermediate metabolizers) experience higher gefitinib exposure and a greater incidence of dose-limiting toxicities such as rash [22]. In one cohort, non-small cell lung cancer patients carrying low-function CYP2D6 genotypes had a significantly increased risk of ≥Grade 2 rash during gefitinib therapy compared to those with normal CYP2D6 activity [22]. This illustrates how diminished metabolic clearance can exacerbate TKI side effects. Although CYP2C9 and CYP2C19 variants have less prominent roles in targeted therapies, they may affect drugs where those enzymes contribute to metabolism (for instance, CYP2C9 helps metabolise certain PI3K/AKT pathway inhibitors). Beyond the CYPs, phase II enzyme polymorphisms can also be relevant: for example, UGT1A128 (Uridine diphosphate glucuronosyltransferase 1 polypeptide A128), a glucuronidation enzyme variant, reduces clearance of the HDAC inhibitor belinostat. Homozygous UGT1A128 patients show increased belinostat exposure, and the FDA recommends a 25% dose reduction in this genotype group to mitigate severe toxicity [23,24].

Alongside metabolism, drug transporters in the ATP-binding cassette (ABC) and solute carrier (SLC) families critically determine the pharmacokinetics of targeted therapies. P-glycoprotein (ABCB1, also known as MDR1) and Breast Cancer Resistance Protein (ABCG2) are efflux pumps that limit oral drug absorption in the gut and actively extrude drugs from cells (including at the blood–brain barrier and tumor cells). Common polymorphisms in ABCB1 can alter its transport function or expression. Notably, the ABCB1 1236C>T, 2677G>T/A, *3435C>T haplotype is associated with reduced P-gp activity [9,10]. In CML, patients carrying ABCB1 1236T or 3435T variants had significantly higher plasma trough levels of the TKI imatinib [25]. This higher exposure translated into improved treatment responses: variant ABCB1 genotypes correlated with higher rates of early molecular response and superior 2-year failure-free survival on imatinib [26]. Diminished P-gp function likely allows greater intracellular retention of the drug, enhancing its anti-leukemic effect. By contrast, patients with high-function P-gp alleles may experience lower drug bioavailability and reduced efficacy of TKIs, underscoring the impact of ABCB1 polymorphisms on therapeutic outcomes.

ABCG2 (BCRP) polymorphisms can similarly influence targeted therapy. The well-studied ABCG2 421C>A variant (Q141K) impairs BCRP transporter activity, leading to increased systemic drug exposure. For instance, cancer patients heterozygous for ABCG2 421C>A had significantly higher steady-state accumulation of the EGFR inhibitors gefitinib and erlotinib compared to wild-type patients [26]. Gefitinib is a substrate of BCRP, and the Q141K variant’s reduction in efflux capacity can elevate plasma and tissue drug levels [26]. While this may improve drug efficacy against tumors, it also raises the risk of toxicities (such as diarrhea or skin rash) due to higher concentrations. Indeed, ABCG2 polymorphisms have been linked to inter-patient differences in TKI tolerance in clinical studies [26]. Other transporter genes in the ABC family, including ABCC2 (MRP2), have variants (e.g., −24C>T, 1249G>A) associated with altered pharmacokinetics of TKIs [24], although their clinical impacts are less documented than ABCB1/ABCG2.

Inherited variations in uptake transporters of the SLC family can also modulate targeted therapy outcomes. Organic cation transporter 1 (OCT1, gene SLC22A1) mediates intracellular uptake of imatinib into leukemic cells [25]. Functional OCT1 activity has been implicated as a determinant of response in CML: polymorphisms like SLC22A1 rs628031 (Met408Val, linked to a splicing defect) are associated with reduced OCT1 function and have been correlated with lower rates of molecular response to imatinib [20]. A meta-analysis confirmed that CML patients carrying certain SLC22A1 variant alleles had significantly worse odds of achieving major molecular response on imatinib therapy [20]. Likewise, that analysis found patients with the CYP3A5*1 allele (expressors) had higher complete cytogenetic response rates, particularly in Asian populations [19], consistent with the notion that active metabolism and uptake can influence drug effectiveness. Polymorphisms in hepatic uptake transporters like OATP1B1 (SLCO1B1) may also alter the clearance of some targeted drugs, as seen with other tyrosine kinase inhibitors and chemotherapeutics [25], although definitive examples in targeted therapy are still emerging.

Organ function also plays a crucial role in drug disposition. Many cytotoxic agents and small-molecule inhibitors rely on hepatic metabolism or renal clearance; impaired liver or kidney function can therefore significantly alter systemic exposure. Population PK modeling and individualized dosing have emerged as essential tools for reducing toxicity in patients with compromised organ function [14,15,27,28].

Therapeutic drug monitoring (TDM) represents a growing strategy to address PK variability. By measuring plasma drug concentrations and adjusting doses accordingly, clinicians can maintain drug exposure within therapeutic ranges, optimizing efficacy while minimizing toxicity. TDM has demonstrated particular utility for narrow therapeutic window drugs, including methotrexate, busulfan, and multiple TKIs [3,7].

TDM can individualize dosing for drugs with narrow therapeutic windows or high pharmacokinetic variability, improving efficacy and safety [29]. For example, 5-fluorouracil (5-FU) dose adjustment guided by plasma levels (target AUC ~20–30 mg·h/L) significantly reduced toxicity and improved response rates compared to standard body-surface dosing [29]. TDM has been shown to optimize outcomes in other cases as well (e.g., high-dose methotrexate rescue, or busulfan in transplant conditioning) by ensuring drug exposure stays within a defined therapeutic range [30]. Many oncologists agree that TDM offers a valuable tool to individualize treatment for patients [30].

However implementing TDM in oncology has also some disadvantages. Not all anticancer drugs have well-defined target concentrations or easy assays. Practical barriers include the cost and availability of drug assays, extra patient visits for blood draws, and delays in dose adjustment while awaiting results. Many clinicians also lack training or guidelines on TDM for newer agents [30]. Furthermore, if only a single drug level is taken, Bayesian dose forecasting can have uncertainty (high “shrinkage”) [31]. In summary, while TDM can reduce under- or overdosing and improve outcomes for certain drugs, it requires resources and evidence-based targets to be effective. It is most justified for drugs with clear exposure–response relationships and narrow safety margins (e.g., busulfan, 5-FU, imatinib) and less useful for drugs without such relationships [30].

### 2.2. Pharmacodynamics and Tumor-Specific Responses

PK, depending on the determination (type of sample), may determine systemic availability and not necessarily availability at the site of effect (within the tumor). On the other hand, PD describes the effects of the drug once it reaches its target. PD encompasses receptor engagement, downstream signaling modulation, and resultant biological consequences on both tumor and normal cells. In oncology, PD variability is largely driven by the molecular heterogeneity of tumors [2,4,9].

Tumors that appear similar histologically may harbor vastly different driver mutations, resulting in divergent drug sensitivities. For example, activating EGFR mutations in non-small-cell lung cancer predict robust responses to EGFR inhibitors, whereas tumors with KRAS mutations are typically resistant. Similarly, BRAF V600E mutations in melanoma indicate likely benefit from BRAF inhibitors, in contrast to wild-type tumors where efficacy is limited [10,12].

Predictive PD biomarkers are instrumental for therapy selection and monitoring. HER2 overexpression in breast cancer identifies patients who benefit from trastuzumab, while PD-L1 (Programmed death-ligand 1) expression serves as a marker to guide checkpoint inhibitor therapy in immuno-oncology [1,5]. Incorporating these biomarkers enables precision therapy, allowing clinicians to target treatments to patients most likely to respond and sparing others from unnecessary toxicity [7,8].

Acquired resistance remains a critical PD consideration. Tumors may initially respond to therapy but subsequently develop secondary mutations or activate alternative pathways. The T790M mutation in EGFR-mutant lung cancer exemplifies this mechanism, conferring resistance to first-generation inhibitors while sensitizing tumors to third-generation agents like osimertinib (OSI) [10,13]. Awareness of such adaptive PD changes informs the development of next-generation inhibitors and rational combination therapies aimed at delaying or overcoming resistance [2,4].

### 2.3. Integrating PK and PD for Precision Oncology

Although often studied separately, PK and PD are most powerful when integrated. The PK/PD relationship quantitatively links systemic drug exposure to therapeutic effect [3,6,14]. In clinical practice, PK/PD modeling increasingly informs dose selection and therapeutic optimization. Checkpoint inhibitors illustrate this integration: population PK/PD analyses have shown that once exposure reaches a threshold, higher doses do not yield additional clinical benefit. Such findings support dose de-escalation strategies, maintaining efficacy while reducing toxicity and healthcare costs [5,9].

Emerging adaptive treatment models incorporate real-time PK/PD data alongside biomarkers such as circulating tumor DNA dynamics, enabling ongoing adjustments to therapy based on patient-specific responses. These adaptive strategies represent a practical step toward true precision oncology, in which treatment evolves continuously in response to both patient physiology and tumor biology [4,11,15].

### 2.4. Individual vs. Population Approaches in PK/PD

In oncology, PK/PD modeling is used at both the population level (to guide drug development and general dosing recommendations) and the individual level (to personalize therapy). In drug development, population PK/PD models are built by pooling data from many patients to characterize typical kinetic and dynamic parameters, along with their variability. This helps define an optimal dosing regimen. For instance, early-phase trials increasingly use PK/PD models to select doses that achieve target exposure or biomarker responses. A population model can integrate data on tumor size changes, tumor markers, and toxicity to find a dose that balances efficacy and safety [32]. One case study showed that a population PK/PD model informed the expansion dose in a Phase I trial by predicting which dose would sufficiently inhibit a tumor signaling pathway in patients [33]. Overall, population modeling allows simulation of “what-if” scenarios (e.g., different dosing schedules, combinations) beyond those tested, thereby guiding rational trial design [32]. Model-based analyses have also been used to support regulatory approvals by evidencing exposure–response relationships for efficacy or safety.

At the individual patient level, PK/PD models underpin model-informed precision dosing. Here, one uses patient-specific data (drug concentrations, biomarkers, genetics) along with a prior population model to optimize that patient’s dose. This is essentially TDM implemented via Bayesian forecasting. A prime example is busulfan dosing in stem cell transplant conditioning. Busulfan has a narrow therapeutic range–too little and the transplant may fail to engraft, too much and severe toxicity (veno-occlusive disease) can occur. Using a population PK model and a few plasma samples from the patient, clinicians adjust the dose to hit a target cumulative AUC (typically about 78–101 mg·h/L) associated with optimal transplant outcomes [30]. Studies showed this model-guided dosing significantly improved event-free survival by keeping exposure in the desired window [34]. Likewise, for oral TKIs like imatinib, sunitinib, and pazopanib, which have high inter-patient PK variability, model-based dose individualization has been proposed. Consensus guidelines recommend, for example, aiming for imatinib trough concentrations ≥1000 ng/mL in chronic myeloid leukemia to ensure adequate response [35], while avoiding very high levels (>3000 ng/mL) that increase toxicity [36]. For sunitinib (used in renal cell carcinoma), studies indicate that maintaining a trough around 50 ng/mL is associated with better progression-free survival [34]. In practice, a blood level is measured and the dose can be escalated or reduced using the model until the patient’s trough falls in the therapeutic range. This individual PK/PD approach, though not yet routine for all TKIs, has shown feasibility and improved tolerability in clinical studies [34,36].

## 3. Targeted Therapies: Variability in PK/PD

Targeted cancer therapies, including small-molecule TKIs and monoclonal antibodies (mAbs), have highly specific mechanisms but show considerable variability in PK/PD profiles across patients [34]. TKIs (e.g., EGFR, ALK, or VEGFR inhibitors) are often oral drugs that undergo hepatic metabolism and are prone to drug interactions and pharmacogenetic differences. Consequently, systemic exposure to a given TKI dose can vary widely between patients, leading to suboptimal dosing in some and excessive toxicity in others [37]. For example, TKIs like pazopanib and sunitinib show >10-fold inter-patient differences in plasma trough levels, raising the risk of under-dosing or over-dosing with fixed regimens [34,37]. This variability has prompted interest in therapeutic drug monitoring (TDM) and model-informed precision dosing for TKIs. Emerging evidence indicates that adjusting TKI doses based on PK/PD targets (e.g., maintaining a minimum plasma concentration associated with response) could improve outcomes in responsive patients [34]. However, routine TDM for all TKIs is not yet standard, as feasibility challenges exist for certain drugs (e.g., very narrow therapeutic windows or unpredictable toxicity profiles) [37]. A recent prospective study identified several targeted oral therapies (cabozantinib, everolimus, etc.) for which TDM was not clinically useful due to either high toxicity at standard doses or consistently adequate drug levels in most patients. These findings underline that while PK variability is common, a personalized dosing approach must be judiciously applied where a clear exposure–response relationship is established [34,37,38].

Monoclonal antibody therapies (e.g., rituximab, trastuzumab, cetuximab) also exhibit PK variability, but their disposition is governed by factors such as target-mediated clearance, immunogenicity, and patient-specific attributes (body size, tumor burden, etc.). mAbs generally have long half-lives and nonlinear clearance at low concentrations due to target binding. Inter-individual variability in mAb PK can be significant–for instance, differences in neonatal Fc receptor function or serum protein levels can alter antibody clearance [38]. Moreover, patients with high tumor antigen expression may clear antibodies faster via target-mediated drug disposition [39]. These PK differences can translate into PD variability; some patients achieve deeper target inhibition or longer response durations than others at the same dose. Unlike TKIs, dosing of mAbs is often weight-based or flat-fixed after initial population PK modeling. Model-based analyses have identified covariates (like body weight, albumin, or anti-drug antibody formation) that explain part of the variability, supporting dose individualization in certain cases [38]. However, broad implementation of personalized mAb dosing is rare, partly because most antibodies have wide therapeutic indices. Instead, biomarker-driven patient selection is critical—e.g., the EGFR antibody cetuximab is only effective in tumors without KRAS mutations, regardless of PK. In summary, targeted therapies demonstrate that “one size fits all” dosing is suboptimal, and accounting for PK/PD variability is key.

For instance, trastuzumab (a mAb against HER2) dramatically improved survival in HER2-positive breast cancer, and immune checkpoint antibodies (e.g., anti-PD-1/PD-L1) have produced durable remissions in multiple tumor types. mAbs’ targeted nature often means fewer off-target effects than conventional chemotherapy, and they can synergize with other treatments (e.g., mAbs that bring chemo directly into cancer cells) [35].

However, the treatment with mAbs also has several limitations. They generally only target cell-surface or extracellular molecules, so cancers without a suitable antigen or with antigen-loss variants may escape treatment [35]. Large antibody molecules also may penetrate solid tumor tissue unevenly, limiting efficacy deep in the tumor mass [35]. mAbs can provoke immune-mediated side effects; common issues include infusion reactions and flu-like symptoms, and some antibodies carry a risk of severe organ toxicities (e.g., cardiomyopathy with anti-HER2 mAb, inflammatory lung disease with some checkpoint inhibitors) [36]. Additionally, cost and complexity are downsides–mAbs are biologics that are expensive to produce and administer (often requiring IV infusions) [16]. Patients may need testing to confirm the target (e.g., HER2 or PD-L1 status) before use, and tumors can develop resistance (such as mutating or shedding the target antigen).

Ongoing approaches include adaptive dosing trials, PK-guided dose escalation for patients with sub-therapeutic levels, and integration of pharmacogenetic data (such as CYP polymorphisms affecting TKI metabolism) to tailor therapy [40]. These strategies aim to ensure each patient maintains drug exposure in the efficacious range while avoiding unnecessary toxicity–a cornerstone of personalized medicine in targeted therapy.

## 4. Immunotherapy: Checkpoint Inhibitors and Chimeric Antigen Receptor T Cells (CAR-T) Cells

Immunotherapies have revolutionized oncology by achieving durable remissions in a subset of patients, but response rates are highly variable. From a PK/PD perspective, immune checkpoint inhibitors (ICIs) like anti-PD-1/PD-L1 or anti-CTLA-4 antibodies present unique characteristics. These mAbs bind immune receptors with high affinity; notably, PK/PD studies reveal a plateau in exposure–response once a threshold receptor occupancy is achieved [40,41]. In fact, relatively low ICI doses can saturate their targets and elicit near-maximal T-cell activation. This explains why, in early trials, pembrolizumab 2 mg/kg had similar efficacy as 10 mg/kg, and flat dosing regimens (e.g., nivolumab 240 mg every 2 weeks) were adopted for convenience [42,43]. The plateau effect and steep dose–response curve at low doses indicate that current approved doses may not always be “optimal” in a PK sense–there is ongoing research into dose de-escalation and extended dosing intervals for ICIs to reduce toxicity and cost without compromising efficacy [41]. For example, many patients maintain responses on pembrolizumab even when doses are lowered or spaced out, reflecting that maximum PD effect can be sustained with less drug once immune activation is achieved [42]. Immunotherapy dosing thus challenges the traditional paradigm that higher exposure yields better tumor kill; instead, once sufficient immune stimulation occurs, additional drug mostly increases adverse events and expense [43]. This recognition has led to trials of shorter ICI courses or lower fixed doses, guided by PK/PD modeling and clinical observations [43]. Future ICI development is likely to incorporate these principles to find minimal effective dosing that achieves durable checkpoint blockade.

Another breakthrough is CAR-T cell therapy, where the “drug” is a living cell product targeting tumor antigens (e.g., CD19 in leukemias). CAR-T therapies introduce entirely new PK/PD concepts: after infusion, CAR-T cells undergo proliferation (expansion) in the patient, distribute to tissues (especially sites of antigen), and can persist long-term as memory cells. The PK of CAR-T is often described by the expansion kinetics (peak and area under the curve of CAR-T cell count in blood) rather than plasma concentration [33]. This expansion correlates with PD outcomes: patients with higher CAR-T expansion tend to achieve deeper remissions but also face higher risk of cytokine release syndrome, an acute toxicity triggered by immune cell activation [44,45]. Thus, the PK/PD relationship for CAR-T is complex and patient-specific–it depends on factors like tumor burden (antigen load can drive more proliferation), immune status, and CAR-T intrinsic design. Biomarker-based patient selection for CAR-T therapies currently centers on tumor antigen expression (only patients whose tumors express the CAR target are eligible) and factors like prior therapies or baseline inflammatory markers that might predict toxicity [21,46]. CAR-T cells can induce profound remissions in otherwise refractory cancers. Notably, in relapsed/refractory B-cell acute lymphoblastic leukemia (ALL), anti-CD19 CAR-T therapy achieved complete remission in ~80% of patients in early trials [41]. This led to the first CAR-T (tisagenlecleucel) being approved in 2017 for pediatric ALL [41], and since then several CAR-T products have been approved for aggressive lymphomas and multiple myeloma. A major benefit is CAR-T’s high specificity and potency: the T cells are “living drugs” that can expand in the body and actively seek out cancer cells. They work independently of MHC presentation, so they can target cancer cells that evade normal T-cell recognition. CAR-T therapy has produced durable responses in many patients, offering hope in diseases like ALL and large B-cell lymphoma where conventional therapy failed. It also exemplifies personalized medicine–each treatment is custom-made from the patient’s own cells.

Given the personalization required, PK/PD modeling and simulation are increasingly used to optimize CAR-T therapy. For instance, mathematical models have been developed to relate CAR-T cell dose and expansion to outcomes, aiding in dose selection for new CAR-T products [46]. Moreover, product-specific attributes (such as CAR construct and T cell phenotype) influence kinetics; understanding these through PK/PD studies helps refine manufacturing for better persistence or controlled expansion. As CAR-T moves into solid tumors, the variability in reaching tumor sites and overcoming the immunosuppressive microenvironment are major PD hurdles. In summary, immunotherapies demand a dual approach: optimize the dose and schedule based on PK/PD science (to achieve sufficient immune activation), and select the right patients using biomarkers to ensure that the activated immune system can indeed recognize and attack the cancer [47]. These principles are vital for maximizing the efficacy of checkpoint inhibitors and CAR-T cells while managing their unique toxicities [46,47].

However, CAR-T therapy comes with serious challenges and risks. Manufacturing is complex and time-consuming; patients must wait weeks for their T cells to be engineered, which is problematic in rapidly progressing disease. The therapy can cause life-threatening acute toxicities, especially cytokine release syndrome (CRS) and neurotoxicity, due to the massive immune activation when CAR-T cells engage tumors [40]. Managing these toxicities requires specialized care (e.g., ICU support, IL-6 blockers like tocilizumab). Another limitation is that most successes have been in blood cancers–CAR-T cells have had limited efficacy in solid tumors so far [40]. Solid tumors can thwart CAR-T cells through poor infiltration (T cells cannot effectively enter the tumor mass) and immunosuppressive microenvironments that deactivate T cells [40]. Tumors may also undergo antigen escape (panel A in the figure), where cancer cells downregulate or lose the target antigen under therapy pressure [37,40]. Additionally, CAR-T cells can attack normal tissues that share the target antigen (on-target/off-tumor effect, panel B), causing severe damage. Beyond medical issues, CAR-T is extremely expensive and available only in specialized centers. In summary, CAR-T therapy is a breakthrough for certain leukemias/lymphomas with remarkable efficacy, but its use is limited by formidable toxicities, high cost, and current ineffectiveness in most solid tumors [48].

## 5. Nanomedicine and Drug Delivery Systems: Overcoming PK Barriers

Many conventional anticancer drugs have suboptimal pharmacokinetics–for example, poor water solubility, rapid clearance, or lack of tumor selectivity–which limit their efficacy and cause systemic toxicity. Nanomedicine approaches aim to overcome these PK barriers by engineering drug delivery systems that improve drug absorption, distribution, metabolism, and excretion profiles. Over the past decade, a variety of nanoparticle-based carriers (liposomes, polymeric nanoparticles, micelles, dendrimers, etc.) have been developed to favorably alter drug biodistribution. A classic example is liposomal doxorubicin (Doxil^®^), which encapsulates doxorubicin in a stealth liposome: this increases the circulating half-life and preferential accumulation in tumor tissue via the enhanced permeability and retention effect, while reducing cardiac exposure and thus lowering cardiotoxicity. Tumor-targeted nanocarriers can achieve higher intra-tumoral drug concentrations than free drug formulations, addressing the challenge of poor tumor uptake [49,50]. Indeed, 15 nanomedicine therapeutics have gained approval globally, highlighting their clinical efficacy in delivering chemotherapy or nucleic acids to tumors (Table 1) [51]. Nanoparticles help drugs evade renal and hepatic clearance, protect them from premature degradation, and can be functionalized with targeting ligands (e.g., antibodies or peptides) to selectively bind tumor cells [52,53,54]. These PK advantages translate into PD benefits: improved therapeutic index and the ability to administer highly potent toxins or gene-silencing molecules that would be too toxic or unstable in free form.

Despite these advances, first-generation nanomedicines faced limitations such as heterogeneous tumor uptake and insufficient penetration into tumor tissue. The tumor microenvironment (TME)–with its abnormal vasculature, high interstitial pressure, and dense stroma–often impedes uniform nanoparticle distribution. To address this, current research is focused on “smart” nanomedicines that are responsive to TME cues. Such stimuli-responsive nanoparticles remain stable in circulation but release their payload upon encountering specific triggers in the tumor (e.g., low pH, certain enzymes, or hypoxia) [35,38]. For instance, pH-sensitive liposomes can unload drugs in the acidic tumor milieu, and enzyme-cleavable coatings allow deeper tissue penetration once activated by matrix metalloproteinases in the TME [74]. These intelligent delivery systems improve the effective drug concentration at the target site and reduce off-target effects, essentially adding a PD layer of control to drug release. Additionally, surface modification of nanoparticles with molecules like folate, transferrin, or antibodies can facilitate receptor-mediated uptake by cancer cells, further enhancing targeting specificity [38]. Recent reviews highlight significant progress in “smart” nanoparticle design, including polymeric nanocarriers that respond to magnetic fields or thermal triggers to concentrate therapy regionally [38]. Another frontier is coupling nanomedicine with immunotherapy–for example, nanoparticle vaccines or drug-loaded nanoparticles that modulate the immune TME (e.g., reprogramming macrophages). By remodeling the TME (reducing immunosuppression and improving tumor perfusion), such approaches tackle PK barriers and can synergize with immunotherapies. Indeed, nanoparticle-mediated delivery of immunomodulators has shown improved immune cell infiltration in tumors and enhanced anti-tumor immunity [43].

Nanomedicine has helped address several long-standing pharmacokinetic (PK) challenges in cancer therapy by redesigning how drugs are delivered, distributed, and metabolized. Traditional small-molecule chemotherapeutics often suffer from poor solubility, rapid clearance, low tumor selectivity, and systemic toxicity. Nanocarriers (liposomes, polymeric nanoparticles, micelles, dendrimers and inorganic nanoparticles) were introduced to overcome these barriers through several mechanisms. Nanomedicine addresses PK obstacles in cancer by improving solubility and stability, prolonging circulation, enhancing tumor selectivity, reducing toxicity, bypassing resistance mechanisms, and enabling controlled or stimuli-responsive release. These advances shift the balance from systemic exposure toward tumor-targeted exposure, a key goal in cancer pharmacotherapy [75,76,77]. The challenge moving forward is to fully realize hierarchical targeting–achieving tissue-, cellular-, and subcellular-level precision in drug delivery [78]. Ongoing innovations such as multi-stage nanoparticles (that change size or charge to penetrate tumors) and AI-guided nanocarrier design are expected to further refine delivery kinetics [79]. With continued development, nanomedicine and advanced drug delivery systems will play a central role in maximizing the therapeutic impact of potent drugs by overcoming the PK barriers that traditionally limited their use.

## 6. Combination Therapies: PK/PD-Based Rationales for Sequencing and Synergy

Combining therapies is a well-established strategy in oncology to enhance efficacy and prevent resistance. However, designing an optimal combination regimen requires careful PK/PD consideration to achieve true synergy (or at least additive benefit) without intolerable toxicity. One key insight is that the sequence and timing of drug administration can profoundly influence the interaction between agents. Drugs given concurrently may exhibit pharmacodynamic antagonism if they interfere with each other’s mechanism or cell-cycle phase action. In contrast, a sequential schedule can separate antagonistic effects and allow each drug to act at the right time. A striking example is the combination of the chemotherapy pemetrexed (PEM) with the EGFR TKI osimertinib in EGFR-mutant lung cancer. Research showed that giving PEM 48 h before OSI yielded much greater tumor cell kill than concurrent dosing [80]. The rationale was PD-driven: PEM causes DNA damage and S-phase arrest in tumor cells, and OSI is most effective if given after this damage accumulates (to trigger apoptosis rather than protect cells in G1) [81]. A 48 h interval allowed PEM’s cytotoxic effects to peak, then OSI was introduced to block EGFR-driven repair and survival signals, resulting in synergistic apoptosis [81]. In contrast, giving both together or in the reverse order led to suboptimal effects, as OSI prematurely halted cell cycling needed for PEM’s action. This illustrates how PK/PD-informed scheduling can turn a potentially antagonistic combo into a synergistic one. Similar sequence-dependent synergy considerations apply to many regimens (administering anti-angiogenic therapy before chemotherapy vs. after, or priming tumors with a DNA demethylating agent before an immune therapy). PK modeling (e.g., using “digital twin” simulations) can be employed to optimize drug intervals and dosing so that drug concentrations and tumor pharmacodynamics align for maximal combined effect [82,83,84].

Beyond scheduling, combination therapy design must account for PK interactions and cumulative toxicity. From a PK standpoint, combining drugs can alter metabolism or clearance–for instance, if one drug inhibits a cytochrome P450 enzyme that clears the other, it may raise the second drug’s exposure. Such interactions may necessitate dose adjustments in combinations. An example is the use of CYP3A4-metabolized TKIs with potent CYP inhibitors; co-administration can boost TKI levels and toxicity, so sequential or lower dosing might be required. On the PD side, overlapping toxicities (e.g., two myelosuppressive agents) often limit combination dosing to sub-maximal levels of each drug. Rational combinations seek to maximize efficacy through complementary mechanisms while minimizing shared toxicities. For instance, combining immunotherapy with targeted therapy might exploit distinct mechanisms (immune activation plus oncogenic pathway inhibition) for additive tumor kill, and some toxicities do not overlap (immune-related vs. pathway-related side effects). However, not all theoretically synergistic mechanisms translate into clinical synergy. A recent analysis of combination trials found that truly supra-additive efficacy (greater than the sum of parts) is quite rare in oncology; many successful combos work via independent action or at best additive effects, especially in immuno-oncology [77,85]. For example, the blockade of PD-1 and CTLA-4 together improves response rates relative to either alone, but this appears to be mostly an additive effect of two independent immune mechanisms, accompanied by compounded toxicity, rather than a novel synergistic effect [86]. The concept of independent drug action posits that each agent in a combo predominantly benefits the subset of patients whose tumors are sensitive to that agent, with limited true interaction. This challenges us to identify combinations where there is negative correlation of resistance (one drug counteracts resistance to the other)—such anti-correlated drug effects can yield synergy by overcoming cross-resistance [87]. An example is the use of alternating or combination therapies to prevent or target resistance mutations: if tumor cells resistant to Drug A become more vulnerable to Drug B (collateral sensitivity), that combo could achieve outcomes impossible with either agent alone [88]. Exploiting such relationships requires deep PD insights and sometimes biomarker-driven adaptive therapy, where treatment is switched based on evolving tumor characteristics [89,90,91].

In practice, rational combination development now often involves PK/PD and quantitative systems pharmacology modeling in preclinical stages to predict optimal pairings and schedules. This model-informed approach helps prioritize combinations that are likely to be synergistic at tolerable doses. Additionally, biomarker strategies are used in combinations: for example, using a DNA damage marker to identify patients who may benefit most from adding a PARP inhibitor to chemotherapy, or monitoring immune cell populations to decide if an immunotherapy should be added to a targeted agent. Combination regimens are most effective when grounded in a clear mechanistic rationale: either simultaneous blockade of parallel pathways, sequential targeting of primary and escape pathways, or modification of the tumor microenvironment to enable a second therapy (such as using an anti-angiogenic to normalize vessels and improve delivery of a cytotoxic agent). Each of these rationales has a PK/PD basis–whether it is drug–drug PK interactions, temporal dynamics of tumor cell kill, or modulation of tissue distribution. Going forward, as new agents like cell therapies and bi-specific antibodies enter combinations, close attention to their PK/PD profiles will be essential. The goal is to design combination therapies that achieve true synergy or at least potent additive effects, guided by PK/PD data and biomarkers, while avoiding simply doubling toxicity. Through combining the abovementioned approaches, the treatment of the patients can be optimized to deliver superior outcomes beyond what single agents could accomplish [74,92,93].

## 7. Challenges and Clinical Implementation

Although PK/PD modeling has revolutionized drug development, its translation into daily oncology practice has been gradual. Practical barriers include the need for clinician education, integration of modeling tools into electronic health systems, and evidence from prospective trials showing improved patient outcomes [94]. Some regulatory agencies have begun including model-informed dosing recommendations in drug labels, signaling a shift toward implementation, but broader uptake requires multidisciplinary collaboration between pharmacologists, clinicians, and data scientists [95].

Dose individualization is central to personalized oncology. Therapeutic drug monitoring has shown clear benefits for certain chemotherapy agents–for example, adjusting 5-fluorouracil doses based on measured plasma concentrations improves response rates and reduces toxicity in colorectal cancer patients, compared to standard dosing. High-dose methotrexate and busulfan are additional examples where routine drug level monitoring is standard of care to achieve precise exposure targets [96]. Adaptive dosing extends beyond TDM to include pharmacogenomic-guided initiation and toxicity-adapted titration strategies. Genetic testing for TPMT (Thiopurine S-methyltransferase) or UGT1A1 (Uridine diphosphate glucuronosyltransferase 1 polypeptide A1) variants, for example, helps prevent severe toxicities with thiopurines or irinotecan, respectively [97]. Emerging concepts such as reinforcement learning–based adaptive dosing are under investigation, aiming to dynamically adjust therapy in real time using longitudinal patient data [5,98].

Pharmacogenetic differences in drug-metabolising enzymes and transporters can be leveraged to personalize cancer treatment. Identifying patients’ genotypes for key genes (CYP3A4/5, CYP2D6, ABCB1, ABCG2, SLC22A1, etc.) can help predict drug exposure, efficacy, and risk of adverse events. For instance, in metastatic renal cell carcinoma treated with sunitinib, the presence of a functional CYP3A5 allele and certain ABCB1 haplotypes was shown to influence toxicity and progression-free survival, respectively [19]. These findings suggest that genotyping such variants prior to therapy could guide TKI selection or dosing to improve outcomes [19]. A tangible example of implementation is UGT1A1 genotyping for patients receiving belinostat: those with UGT1A128/28 are started at a reduced dose to prevent life-threatening toxicity [23]. As targeted therapy options expand, so does the recognition that one dose does not fit all. Incorporating polymorphism data into treatment planning can help optimize drug choice and dosing for each patient, enhancing efficacy while minimizing toxicity [19].

While personalized medicine offers clear clinical benefits, its economic and ethical implications are substantial. Targeted therapies and immunotherapies are often associated with high costs, raising concerns of sustainability and equitable access [99]. The expense of genomic testing and biomarker assays further amplifies disparities, as patients in low-resource settings or without comprehensive insurance may be excluded from these advances [100]. Ethically, the principles of autonomy, beneficence, and justice must be balanced. Personalized approaches honor individual patient profiles but risk widening gaps between populations if access remains unequal. Issues of financial toxicity, patient privacy regarding genomic data, and the management of incidental findings all represent ongoing ethical challenges [101,102].

Key barriers include the high cost of biomarker testing, insufficient laboratory infrastructure, and inconsistent reimbursement policies. Operational inefficiencies such as long turnaround times or lack of clinician awareness further limit the use of precision diagnostics [103]. Regulatory processes also remain complex, particularly around the co-development of targeted drugs and companion diagnostics, and the evidentiary requirements for rare subgroups [104,105]. Addressing these barriers will require harmonized policies, investment in infrastructure, and educational initiatives to ensure that the promise of personalized medicine becomes accessible to all patients [102]. Ensuring that advances are implemented responsibly—guided by pharmacological insights, supported by real-world evidence, and evaluated within ethical and economic frameworks—will be critical to realizing the full potential of personalized cancer medicine.

## Figures and Tables

**Table 1:** FDA/EMA-Approved Nanomedicine Therapeutics for the Treatment of Cancer.

Therapeutic (Approval Year)	Nanocarrier Type	Active Drug	Nanoparticle Size (nm)	Administration Route	Target Cancer Type (Indication)
**Doxil** (CAELYX^®^)–Pegylated Liposomal Doxorubicin (1995 FDA; 1996 EMA)	PEGylated STEALTH^®^ liposome [55]	Doxorubicin	~100 nm [55]	Intravenous (IV) infusion	AIDS-related Kaposi’s sarcoma, platinum-refractory ovarian cancer, multiple myeloma (with bortezomib) [56].
**DaunoXome**–Liposomal Daunorubicin (1996 FDA)	Conventional (non-PEG) liposome [57]	Daunorubicin (citrate salt)	~45 nm (mean diameter) [57]	IV infusion	First-line cytotoxic therapy for advanced HIV-associated Kaposi’s sarcoma [57].
**Myocet**–Non-PEG Liposomal Doxorubicin (2000 EMA)	Non-PEG multilamellar liposome (egg PC/Chol) [58]	Doxorubicin	150–250 nm [58]	IV infusion	First-line treatment of metastatic breast cancer (in combination with cyclophosphamide) [59].
**DepoCyt** (DepoCyte)–Liposomal Cytarabine (1999 FDA)	Multivesicular liposome (DepoFoam^®^ technology) [60]	Cytarabine	3–30 µm [60] (multi-vesicular)	Intrathecal injection	Lymphomatous meningitis (neoplastic meningitis in lymphoma/leukemia).
**Mepact**–Liposomal Mifamurtide (L-MTP-PE) (2009 EMA)	Multilamellar liposome (<100 nm) [60]	Mifamurtide (muramyl tripeptide phosphatidylethanolamine)	<100 nm [61]	IV infusion (after reconstitution)	Non-metastatic resectable high-grade osteosarcoma (adjunct to surgery and chemotherapy in patients 2–30 y) [61].
**Marqibo**–Vincristine Sulfate Liposome (2012 FDA)	Sphingomyelin–cholesterol liposome (optisome) [62]	Vincristine sulfate	~100 nm [63]	IV infusion	Relapsed or refractory Philadelphia–negative acute lymphoblastic leukemia (adult ALL, ≥2 prior lines) [62].
**Onivyde**–PEGylated Liposomal Irinotecan (2015 FDA; 2016 EMA)	PEGylated nanoliposome (DSPC/Chol/PEG-DSPE) [63]	Irinotecan (topoisomerase I inhibitor)	~110 nm [64]	IV infusion	Metastatic pancreatic adenocarcinoma (after gemcitabine, in combination with 5-FU/leucovorin) [63].
**Vyxeos** (CPX-351)–Liposomal Daunorubicin/Cytarabine (2017 FDA; 2018 EMA)	Fixed-ratio (5:1) co-encapsulated liposome [64]	Daunorubicin + Cytarabine	~100 nm [64]	IV infusion	Newly diagnosed therapy-related AML or AML with myelodysplasia-related changes (“secondary” AML in adults) [65].
**Abraxane** (ABI-007)–Albumin-Bound Paclitaxel (2005 FDA; 2008 EMA)	Albumin-bound nanoparticle (nab-paclitaxel) [66]	Paclitaxel	~130 nm [66]	IV infusion	Metastatic breast cancer; locally advanced or metastatic non–small cell lung cancer; metastatic pancreatic cancer [67].
**Oncaspar**–PEGylated L-asparaginase (Pegaspargase) (1994 FDA; 2007 EMA)	PEG–protein conjugate (monomethoxy-PEG covalently linked to L-asparaginase) [67]	L-Asparaginase enzyme (*E. coli*–derived)	N/A (macromolecule)	IV infusion (or IM)	Acute lymphoblastic leukemia (ALL)–used in multi-agent chemotherapy regimens (including in patients with hypersensitivity to native asparaginase) [56].
**Apealea**–Micellar Paclitaxel (XR17 nanomicelles) (2018 EMA)	Cremophor-free micellar formulation [68] (sodium oleate/retinoid-based micelles)	Paclitaxel	~20–60 nm (est.)	IV infusion	Platinum-sensitive recurrent ovarian cancer (and primary peritoneal or fallopian tube cancer) in combination with carboplatin [69].
**Hensify** (NBTXR3)–Hafnium Oxide Nanoparticles (2019 CE Mark EU)	Inorganic crystalline nanoparticle (radioenhancer) [56]	N/A (no drug, physical enhancer)	~50 nm (crystalline)	Intratumoral injection (pre-radiotherapy)	Locally advanced soft tissue sarcoma (in combination with radiotherapy) [56,70].
**NanoTherm**–Iron Oxide Magnetic Nanoparticles (2010/2013 CE Mark EU)	Inorganic superparamagnetic iron oxide nanoparticles [69] (aminosilane-coated)	N/A (no drug, thermal ablation agent)	~15 nm cores (clustered)	Intratumoral injection (with alternating magnetic field)	Refractory glioblastoma (brain tumor)–device-assisted thermal ablation; also under investigation for prostate and pancreatic cancers [56,67].
**Kadcyla**–Ado-trastuzumab Emtansine (T-DM1 ADC) (2013 FDA/EMA)	Antibody–drug conjugate (humanized anti-HER2 IgG1 linked to DM1 cytotoxin) [67]	Trastuzumab–emtansine (maytansine derivative)	~10–15 nm (antibody)	IV infusion	HER2-positive breast cancer (metastatic, post-trastuzumab/taxane; also approved for adjuvant therapy in residual disease) [67,71].
**Fyarro** (ABI-009)–Albumin-Bound Sirolimus (nab-rapamycin) (2021 FDA)	Albumin-bound nanoparticle (mTOR inhibitor) [72]	Sirolimus (rapamycin)	~100 nm (similar to nab-paclitaxel)	IV infusion	Locally advanced unresectable or metastatic malignant PEComa (perivascular epithelioid cell tumor) [73].

## Data Availability

No new data were created or analyzed in this study. Data sharing is not applicable to this article.

## Abbreviations

The following abbreviations are used in this manuscript:

PK	Pharmacokinetic
PD	Pharmacodynamic
CAR-T	Chimeric Antigen Receptor T cells
CML	Chronic Myeloid Leukemia
NSCLC	Non-Small-Cell Lung Cancer
OSI	Osimertinib
TKIs	Tyrosine Kinase Inhibitors
TDM	Therapeutic drug monitoring
mAbs	monoclonal antibodies
ICIs	immune checkpoint inhibitors
PD-L1	Programmed Death-Ligand 1
irAEs	immune-related adverse events
PEM	Pemetrexed
